# Tick receptor for outer surface protein A from *Ixodes ricinus* **—** the first intrinsically disordered protein involved in vector-microbe recognition

**DOI:** 10.1038/srep25205

**Published:** 2016-04-26

**Authors:** Anna Urbanowicz, Dominik Lewandowski, Kamil Szpotkowski, Marek Figlerowicz

**Affiliations:** 1 Institute of Bioorganic Chemistry, Polish Academy of Sciences, Poznan, 61-704, Poland; 2 Institute of Computing Science, University of Technology, Poznan, 60-965, Poland

## Abstract

The tick receptor for outer surface protein A (TROSPA) is the only identified factor involved in tick gut colonization by various *Borrelia* species. TROSPA is localized in the gut epithelium and can recognize and bind the outer surface bacterial protein OspA via an unknown mechanism. Based on earlier reports and our latest observations, we considered that TROSPA would be the first identified intrinsically disordered protein (IDP) involved in the interaction between a vector and a pathogenic microbe. To verify this hypothesis, we performed structural studies of a TROSPA mutant from *Ixodes ricinus* using both computational and experimental approaches. Irrespective of the method used, we observed that the secondary structure content of the TROSPA polypeptide chain is low. In addition, the collected SAXS data indicated that this protein is highly extended and exists in solution as a set of numerous conformers. These features are all commonly considered hallmarks of IDPs. Taking advantage of our SAXS data, we created structural models of TROSPA and proposed a putative mechanism for the TROSPA-OspA interaction. The disordered nature of TROSPA may explain the ability of a wide spectrum of *Borrelia* species to colonize the tick gut.

Ticks belonging to the *Ixodidae* family are considered the most important vectors of numerous pathogens of humans and animals. When these parasites feed on vertebrate blood, various microorganisms enter and colonize their bodies. The most common species carried by ticks include *Borrelia, Anaplasma, Babesia,* and *Rickettsia*. The mechanism by which this wide spectrum of microbes can colonize the tick gut remains elusive^1^. To date, only one tick-encoded protein that is involved in its colonization by pathogens, the tick receptor for outer surface protein A (TROSPA), has been identified. TROSPA was shown to be present in the tick gut and to interact with *Borrelia* spirochetes’ outer surface protein A (OspA)^2^. As a result, the spirochetes can remain associated with the tick gut epithelium. Thus, TROSPA-OspA binding is considered the first and mandatory step of the tick colonization process. The latest reports have suggested that TROSPA from *Rhipicephalus microplus*, which shares high homology with its counterpart from the *Ixodes* genus, might also be involved in the colonization of the tick by another pathogenic microorganism, *Babesia bigemina*^3^,^4^. Unfortunately, the physiological role of TROSPA, the nature of its interactions with OspA, and its spatial structure remain unknown. Several observations have suggested that TROSPA is subjected to posttranslational modifications. However, a 16-kDa TROSPA from *I. scapularis* and *I. ricinus* produced in a bacterial system was also shown to be capable of binding OspA^2^,^5^. Thus, posttranslational modifications of TROSPA are dispensable for these interactions. TROSPA is predicted to contain a transmembrane helix at its N-terminus, and this helix is most likely not involved in TROSPA-OspA interactions because a TROSPA mutant called TROSPA_NΔ44 that lacks its N-terminal transmembrane domain (44 amino acids located at the N-terminus) binds OspA to the same degree as the full-length protein^5^. Thus, TROSPA-OspA interactions might be electrostatic in nature because at pH 7, the overall charge of the TROSPA protein is −12 and that of OspA is +5. Indeed, it was shown that substitution of the negatively charged amino acid residues with neutral amino acid residues in recombinant TROSPA partially reduces its capacity to bind OspA^5^.

Based on the aforementioned observations, we hypothesized that the polypeptide chain of TROSPA is at least partially unstructured and that TROSPA belongs to the family of intrinsically disordered proteins (IDPs). Studies conducted over the last decade showed that approximately one-third of all eukaryotic proteins contain disordered regions^6^. IDPs perform various functions due to their ability to recognize and selectively bind multiple partners^7^,^8^,^9^. TROSPA from ticks belonging to *Ixodidae* has been reported to interact with at least two pathogen-derived effectors^2^,^3^,^4^. Other IDP-specific features that have been observed for TROSPA include a high content of polar and negatively charged amino acid residues and abnormal electrophoretic mobility during SDS-PAGE^2^,^5^,^10^,^11^. Here, we present ample evidence showing that TROSPA is indeed a new member of the IDP family and is the first example of an extracellular IDP engaged in the colonization of a vector by pathogenic microbes.

## Results

### Bioinformatics search for disordered regions in TROSPA

**Figure 1:**
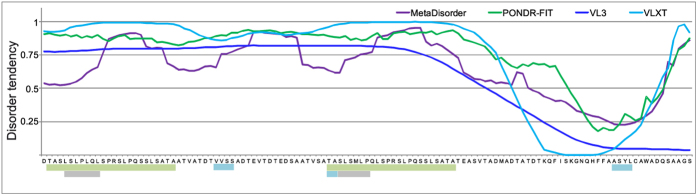
Analysis of TROSPA_NΔ44 secondary structure using online disorder prediction tools and molecular recognition features (MoRFs) prediction tools. Local disorder is shown as a function of TROSPA_NΔ44 amino acid sequence. Each curve represents the results from a different disorder predictor. All residues with a disorder tendency exceeding 0.5 were considered to be disordered. The colored bars below the *x* axis mark sequence motifs with potential functional importance: predicted MoRFs [5–11: LSLPLQL and 56–61: SLSMLP according to ANCHOR (marked with gray bar) and 33–36: VVSS, 54–55: TA, 107–110: ASYL according to MoRFpred (marked with blue bar)] and sequence duplications [2–25: TASLSLPLQLSPRSLPQSSLSATA and 54–77: TASLSMLPQLSPRSLPQSSLSATA (marked with green bar)].

To determine whether TROSPA contains any disordered regions, its sequence was compared with the sequences of IDPs included in the DisProt database^12^. As a result, we found that TROSPA did not exhibit significant similarity to any previously identified IDP (some regions of TROSPA displayed an identity as low as 30% to short regions present in other IDPs). Interestingly, TROSPA also was not similar to any protein whose structure had been determined (according to NCBI BLAST analysis, no known protein structure exhibits 20% or higher sequence identity with TROSPA). In the next stage, TROSPA was examined with disorder predictors available from the GeneSilico MetaDisorder and DisProt websites^13^,^14^. The results of all predictions were consistent and showed a long disordered region spanning the entire sequence, except for approximately 25–30 amino acid residues from the C-terminus (Fig. 1). An earlier comparative analysis of IDPs revealed that typical members of this protein family are often characterized by low mean hydropathy, high net charge per residue (NCPR) as well as fraction charged residues (FCRs) and an extremely low or high isoelectric point (pI)^11^,^15^,^16^. These parameters were all determined for TROSPA and its mutant devoid of the N-terminal transmembrane domain, TROSPA_NΔ44. The calculated values of the mean hydropathy (TROSPA 0.5268 and TROSPA_NΔ44 0.4999), NCPR (TROSPA 0.077 and TROSPA_NΔ44 0.065) and FCR (TROSPA 0.135 and TROSPA_NΔ44 0.14) were not typical for IDPs. These values classified TROSPA as an ordered or globule-forming protein on charge-hydropathy plots^15^,^16^. Only the predicted pI values (3.83 for TROSPA and 4.03 for the TROSPA_NΔ44) were typical of IDPs. Overall, the *in silico* analyses indicated that TROSPA displays at least some features that are characteristic of IDPs. IDPs often contain important functional elements, such as repeated sequences and short transiently or permanently structured regions located within longer intrinsically disordered regions called molecular recognition features (MoRFs)^17^,^18^,^19^,^20^,^21^,^22^,^23^. Interestingly, few such motifs were found in TROSPA. The most remarkable duplicated sequence motif included positions 2–25 and 54–77 of the TROSPA_NΔ44 polypeptide chain; these sequences differ by only one amino acid. We also employed two MoRFs predictors to search for the location of probable regions of ligand binding in TROSPA, and this analysis identified a few such regions, which are shown in Fig. 1.

### Physicochemical properties of TROSPA

**Figure 2:**
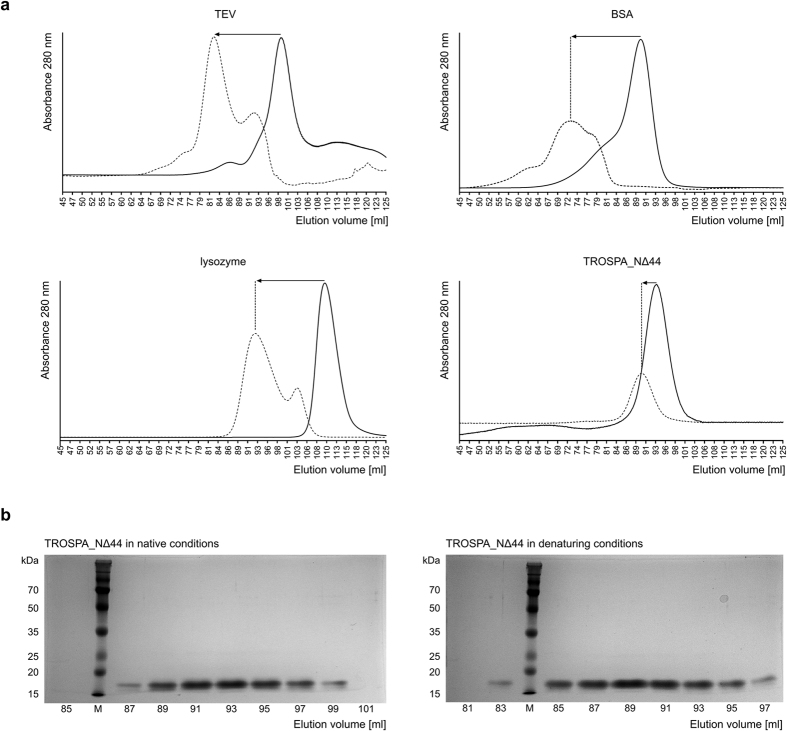
Size-exclusion chromatography elution profile of TROSPA_NΔ44. (**a**) TROSPA_NΔ44 (M_m_ = 12.5 kDa), bovine serum albumin (BSA, M_m_ = 66.5 kDa), TEV protease (M_m_ = 27 kDa) or lysozyme (M_m_ = 14.3 kDa) were separated by SEC on HiLoad 16/60 Superdex 200 pg column, under native (regular line) or denaturing conditions (dashed line). The difference between the elution volumes in native versus denaturing conditions of the analyzed proteins is marked with black arrows. (**b**) SDS-PAGE analysis of fractions from across the elution peaks after SEC of TROSPA_NΔ44 (M – protein molecular weights marker).

IDPs are characterized by a number of specific physicochemical features that clearly distinguish them from proteins that adopt a stable spatial structure, including specific amino acid composition, larger hydrodynamic dimensions compared with typical globular proteins with corresponding molecular mass (M_m_), a low secondary structure content and multi-conformer status^24^,^25^. Thus, they can be differentiated by applying standard electrophoretic, chromatographic or spectroscopic methods that are used in instrumental analysis of bioorganic compounds. Unfortunately, TROSPA contains a highly hydrophobic putative transmembrane domain at its N-terminus^5^. Consequently, the full-length protein shows low solubility and a tendency to aggregate in aqueous solution. To avoid these problems, instead of using the full-length TROSPA, we used TROSPA_NΔ44. As mentioned above, this mutant can bind OspA from *B. burgdorferi* to at least the same degree as TROSPA^5^. The first method that was applied was SDS-PAGE, which is routinely used to estimate the M_m_ of proteins. Because of their specific amino acid composition, IDPs are known to bind less SDS than globular proteins; therefore, their M_m_ observed in SDS-PAGE is approximately 1.2- to 1.8-fold higher than the value calculated from the amino acid sequence or measured by mass spectrometry^10^,^26^. The mobility of the 165-amino acid TROSPA and that of the 121-amino acid TROSPA_NΔ44 in SDS-PAGE corresponded to M_m_ values of approximately 21 kDa^5^ and 16.5 kDa, respectively (Fig. 2), whereas the M_m_ values calculated from their sequences and measured by mass spectrometry were 16.5 kDa^5^ and 12.5 kDa, respectively (for TROSPA_NΔ44 mass spectrum, see Supplementary Fig. S1). Thus, the apparent M_m_ of these proteins was 1.3-fold higher than the calculated M_m_.

Next, we assessed the molecular mass-volume relationship by fractionating TROSPA_NΔ44 and three other well-known proteins, which served as M_m_ markers for the size-exclusion chromatography (SEC) in native conditions. We observed that TROSPA_NΔ44 eluted as a 50–60 kDa protein, immediately behind the bovine serum albumin monomer (M_m_ = 66.5 kDa) and well ahead of Tobacco Etch Virus (TEV) protease (M_m_ = 27 kDa). Compact molten globule-type IDPs were previously shown to elute at an apparent M_m_ that is approximately 2-fold higher than the calculated value, whereas more extended, random coil–type IDPs elute at an apparent M_m_ that is 4- to 6-fold higher than the calculated value^27^ (Fig. 2). Thus, the obtained results suggest that TROSPA is characterized by a highly extended hydrodynamic volume resembling that of coil-type IDPs^28^. An enlarged molecular volume of a protein can also be interpreted in terms of an oligomeric state. To rule out this scenario, we performed SEC under denaturing and reducing conditions (Fig. 2). In this experiment, elution peaks of all analyzed proteins except TROSPA_NΔ44 shifted approximately 17 ml, as compared to SEC in native conditions, whereas for TROSPA_NΔ44 the difference was only 4 ml. This observation indicates that only a small degree of TROSPA_NΔ44 denaturation took place, in contrast to the other analyzed proteins. Thus, the enlarged molecular volume of TROSPA_NΔ44 likely results from its random-coil shape and not from its oligomeric state. We also determined the hydrodynamic dimensions of TROSPA_NΔ44 using dynamic light scattering (DLS) measurements. The hydrodynamic radius (R_h_) of TROSPA_NΔ44 increased from 2.9 (SD ± 0.31) nm to 3.5 (SD ± 0.61) nm over a temperature range of 4–80 °C, which corresponds to an M_m_ of 41–63 kDa for the globular protein^28^. Selected DLS data can be found at Supplementary Fig. S2.

**Figure 3:**
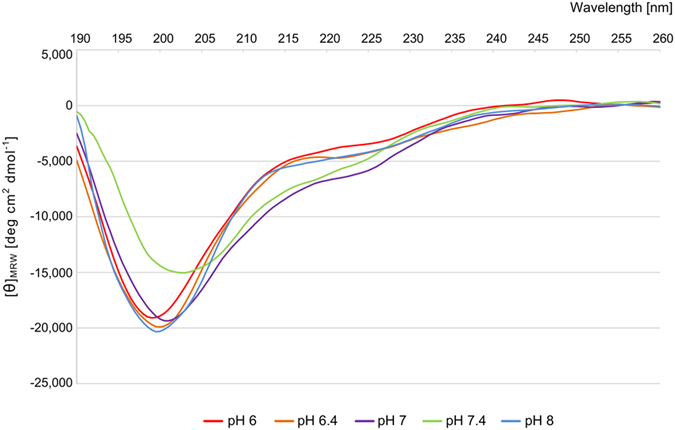
UV CD spectrum of TROSPA_NΔ44 over pH range of 6–8 at 37 °C. The ordinate is expressed as the mean residue ellipticity [θ]_MRW_.

The structural properties of IDPs are strongly influenced by environmental modifications^29^. TROSPA-OspA binding occurs in the tick gut after the initiation of feeding. The pH of the tick gut content at this point is approximately 7.4 and decreases to 6.8 after a few days. The temperature during feeding increases from approximately 20 °C (atmosphere temperature) to 37 °C (host temperature). Moreover, *Borrelia* spirochetes produce OspA protein when grown *in vitro* at pH 7.4 over a temperature range of 23–37 °C^1^,^30^. Thus, the TROSPA-OspA interaction likely occurs at approximately pH 7.4 and over temperature range of 23–37 °C. To monitor the response of TROSPA_NΔ44 to a changing environment, we analyzed its secondary structure as a function of pH and temperature via circular dichroism (CD) measurements. These measurements were performed in phosphate buffer over a pH range of 6–8 and a temperature range of 5–80 °C. Each spectrum exhibited a strong minimum of the mean residue ellipticity [θ]_MRW_ at approximately 195 nm (less than −20,000 deg cm^2^ dmol^−1^) (Fig. 3). Such a spectrum shape is characteristic of IDPs^25^. The contents of specific secondary structures were estimated from the CD spectra using the CONTIN/LL algorithm available from the DichroWeb online server^31^,^32^ (Supplementary Table S1). Interestingly, the results indicated that the secondary structure content in TROSPA_NΔ44 was highest at pH 7.4 and over a temperature range of 35–40 °C, reaching almost 50%, whereas this value was approximately 30% at pH 7, 25% at pH 8 and 20% at pH 6.4 over the same temperature range (Supplementary Table S1). The observed increase in the secondary structure content at pH 7.4 included both α-helices and β-strands, whereas turns were affected to a lesser extent.

**Figure 4:**
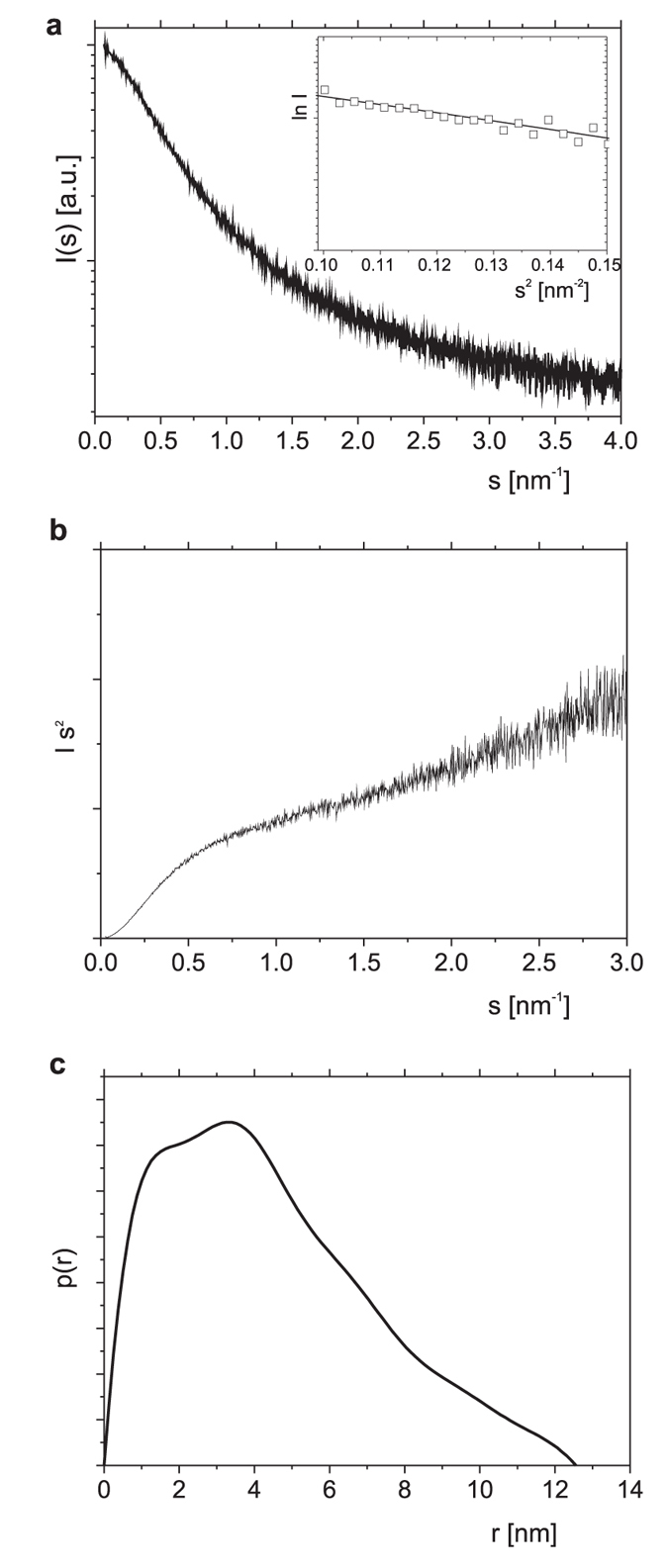
SAXS data on TROSPA_NΔ44. (**a**) The experimental X-ray scattering data plotted as a function of the scattering angle for TROSPA_NΔ44. The Guinier plot is shown in the inset. (**b**) Kratky plot for TROSPA_NΔ44. (**c**) Pair-distance distribution function for TROSPA_NΔ44.

Information concerning the degree of ordering and the molecular dimensions of proteins can be obtained from small angle X-ray scattering (SAXS) measurements. The SAXS profile of a given molecule is the average of all scattering curves measured for all of its conformers. SAXS data are usually displayed as a Kratky plot, which represents the dependence of the scattering intensity (Is^2^) on the scattering angle (s). The Kratky plots of compact globular proteins are typically bell-shaped and usually exhibit a clear maximum. A slight upward slope at higher scattering angles is characteristic of semi-folded proteins, whereas Kratky plots of proteins classified as intrinsically disordered are characterized by a steep upward slope at higher scattering angles (s)^29^,^33^,^34^,^35^,^36^. The Kratky plot obtained for TROSPA_NΔ44 in solution at pH 8 clearly indicates that this protein belongs to the IDP family (Fig. 4b). The radius of gyration (R_g_) of TROSPA_NΔ44 was calculated directly from the SAXS curves using a classical Guinier approximation. The Guinier plot used to determine the R_g_ value (3.73 nm) is shown in the inset in Fig. 4a. The value of R_g_ is larger than that for globular proteins with similar M_m_ values, which suggests the extended dimensions of this molecule^29^,^33^. The pair-distance distribution function, p(r), is a histogram of all inter-atomic distances (r) within the protein, which provides information on the size and overall shape of the molecule. The histogram shows the mean particle size, which is represented by the radius of gyration (R_g_), and the maximal intra-molecular distance (D_max_). The D_max_ of TROSPA_NΔ44 in solution reached a value of 12.56 nm (Fig. 4c), and the calculated p(r) function was asymmetric, which indicated the elongated shape of the protein^29^,^34^.

**Figure 5:**
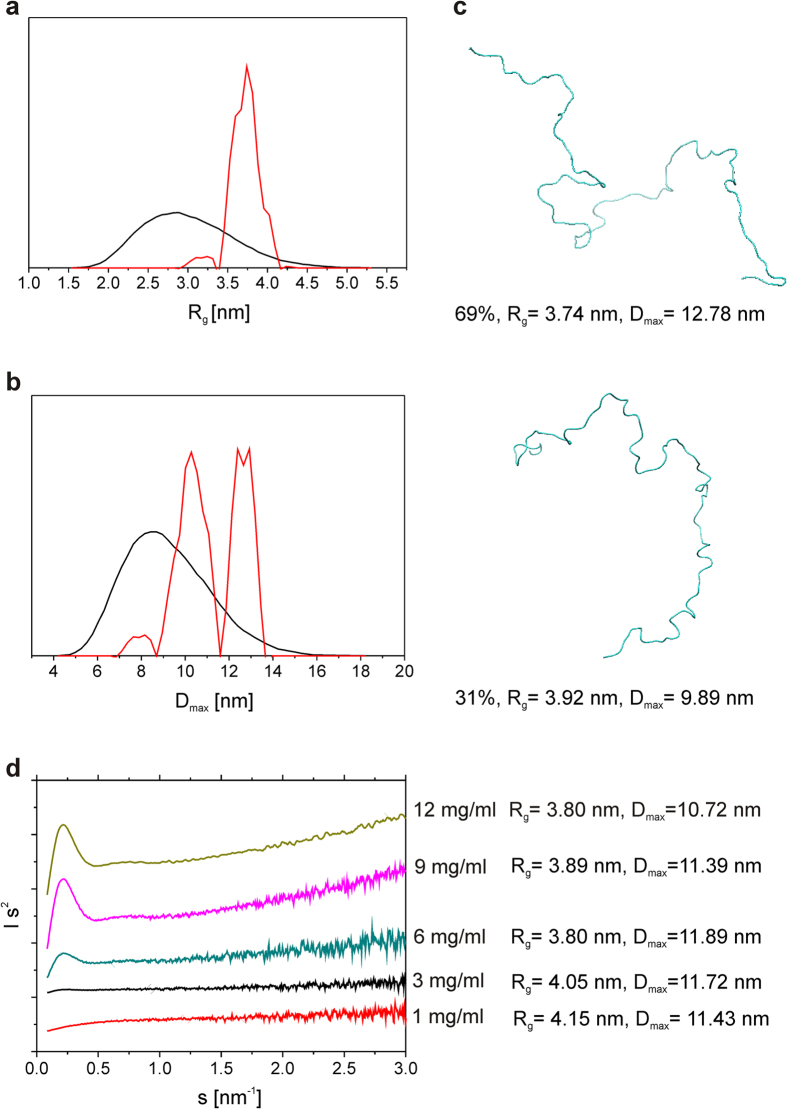
SAXS data processed by EOM for TROSPA_NΔ44 at increasing concentrations. (**a**) R_g_ and (**b**) D_max_ distributions for TROSPA_NΔ44 at 6 mg/ml concentration plotted as functions of frequency (arbitrary units) indicate the bimodal character of the protein population. The red curves represent the distribution that best fits the SAXS data. The black curves represent the theoretical distribution of R_g_ and D_max_ values for an entire polypeptide chain that can form all possible conformations. (**c**) The bimodal population of TROSPA_NΔ44 at the concentration of 6 mg/ml consists of two conformers. (**d**) Kratky plots for TROSPA_NΔ44 at increasing concentrations and the corresponding averaged R_g_ and D_max_ parameters.

The structural parameters of IDP are often sensitive to the concentration of the surrounding macromolecules. At higher concentrations (molecular crowding conditions), some IDPs react with compaction or extension, whereas the others remain unaffected^37^,^38^,^39^,^40^,^41^. The impact of crowding on cellular proteins has been studied at protein/crowder concentrations of up to 300 mg/ml, which mimics the conditions in the cytoplasm^41^. However, TROSPA is an extracellular protein, and we assumed that the analysis of its concentration-structure relationship should be performed under conditions resembling those of gastric or interstitial fluids (protein concentrations of approximately 1 mg/ml and up to 20 mg/ml, respectively)^42^,^43^. The ensemble optimization methodology (EOM) is often used to characterize intrinsically disordered proteins in solution^29^,^44^. EOM creates a set of all possible conformers of a molecule that together represent the observed scattering profile and allows its characterization. We applied EOM to process experimental SAXS data collected for TROSPA_NΔ44 solution at pH 8 and at five different concentrations: 1, 3, 6, 9 and 12 mg/ml. This analysis revealed that a few (2 to 4) conformational populations of the protein (regarding both R_g_ and D_max_) are present at each concentration. Figure 5a,b provide selected data concerning the conformational populations of TROSPA_NΔ44 at a concentration of 6 mg/ml. The entire dataset generated by EOM is shown in Supplementary Fig. S3 and in Supplementary Table S2. The multimodal distribution of the TROSPA_NΔ44 population indicates that intramolecular interactions in the protein force the particular shape of the conformers. This result supports the aforementioned CD spectroscopy results concerning the low secondary structure content. Exemplary EOM-generated conformers of TROSPA_NΔ44 at concentration of 6 mg/ml are shown in Fig. 5c. The average R_g_ and D_max_ values assessed for the most representative EOM-generated conformers are significantly higher than the values assessed for the theoretical population of the protein forming all possible structures, indicating the elongated shape of the protein (Fig. 5a,b)^45^. The Kratky plots and the corresponding EOM-generated parameters (the arithmetic mean of R_g_ and D_max_) for each TROSPA_NΔ44 concentration are presented in Fig. 5d. Interestingly, the increasing protein concentration is coupled with the appearance of a maximum on the Kratky plot, accompanied by decreases in the R_g_ and D_max_ values. Also the R_g_ estimated from Guinier approximation decreases with the increase of concentration (Supplementary Table S2 and Supplementary Fig. S4). This result indicates that TROSPA_NΔ44 becomes more compact at higher concentrations^29^. The observed phenomenon is not caused by aggregation or interparticle interference which is supported by the linearity of Guinier and I_0_ versus concentration plots and molecular masses estimations (see Supplementary Table S2 and Supplementary Fig. S4). Thus, the compaction phenomenon is caused by the entropic effect of steric exclusion^37^,^38^,^39^,^40^,^41^.

### TROSPA-OspA interaction

**Figure 6:**
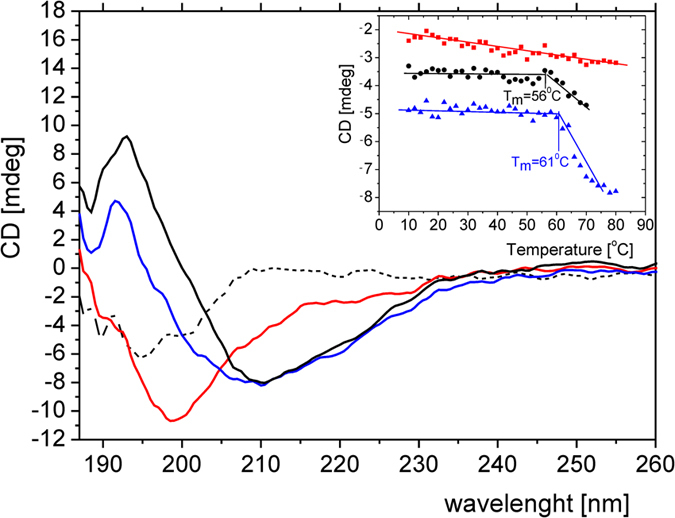
UV CD spectra of TROSPA_NΔ44, OspA_NΔ6 and their complex. The TROSPA_NΔ44 spectrum is in red, the OspA_NΔ6 spectrum is in black, the complex spectrum is in blue and the differential TROSPA_NΔ44 spectrum is represented by a dashed line. The phase transitions of TROSPA_NΔ44, OspA_NΔ6 and their complex during thermal melting are reflected by the change in the ellipticity, which was monitored at 222 nm and is shown in the inset.

The only currently known biological activity of TROSPA relies on OspA recognition and binding. Earlier, we used antibody-based tests to show that TROSPA_NΔ44 retains the ability to bind OspA^2^,^5^. Here, we present preliminary studies of the TROSPA-OspA complex in solution. Previous structural studies of OspA from *Borrelia burgdorferi sensu stricto*, including the crystallographic, SAXS and NMR approach, have examined the protein devoid of the first 17 amino acid residues at its N-terminus^46^,^47^,^48^,^49^. We used truncated OspA from *Borrelia burgdorferi sensu stricto* devoid of the first 6 amino acid residues at its N-terminus, and this truncated mutant is termed OspA_NΔ6. The full-length form of this protein is lipidated at its N-terminus, which results in low solubility and a tendency to aggregate in aqueous solution^5^,^46^. We analyzed a spectrum of deletion mutants of both TROSPA and OspA proteins and found that the above-mentioned modifications were the least indispensable to obtain proteins without a tendency to aggregate. The process of protein-protein recognition and binding is usually accompanied by alterations of the protein secondary structures. Thus, we again employed CD spectroscopy to gain insight into the structural changes that occur in response to TROSPA_NΔ44-OspA_NΔ6 interaction. Earlier, OspA was shown to adopt a stable structure in solution that consists mainly of β-sheets^46^,^47^,^48^. Accordingly, we assumed that the vast majority of structural changes induced upon TROSPA_NΔ44-OspA_NΔ6 binding, especially the appearance of α-helical structures, should occur in TROSPA_NΔ44. Thus, subtracting the OspA_NΔ6 CD spectrum from the CD spectrum of the complex should reveal differences between the secondary structure contents of unbound and bound TROSPA_NΔ44. We recorded CD spectra for TROSPA_NΔ44, OspA_NΔ6 and the mixture of TROSPA_NΔ44-OspA_NΔ6 (molar ratio 1:1) in phosphate buffer at 25 °C and pH 7.4. As shown in Fig. 6, the CD spectra of unbound and bound TROSPA_NΔ44 (obtained by subtracting the OspA_NΔ6 spectrum from the complex CD spectrum) were significantly different. The complexed TROSPA_NΔ44 exhibited a higher level of α-helical organization, as reflected in the negative ellipticity values at 222 nm and 208 nm and a positive value at 190 nm in the CD differential spectrum. To confirm our hypothesis, we thermally melted the complex over the temperature range of 5–80 °C and monitored the changes in secondary structure via CD measurements. We observed a linear correlation between temperature and ellipticity at 222 nm for unbound TROSPA_NΔ44, which indicated a lack of cooperative unfolding that is characteristic of a disordered polypeptide chain (Fig. 6, inset). By contrast, the changes in ellipticity at 222 nm during the thermal melting of OspA_NΔ6 and the complex indicated a phase transition at 56 °C for OspA_NΔ6 and 61 °C for the complex. This result suggests that the TROSPA_NΔ44-OspA_NΔ6 complex contained more α-helical structures than either of the unbound proteins. This finding is supported by the analysis of the CD spectra using algorithms accessible at the DichroWeb online server, which revealed a decrease in the estimated content of disordered regions in the TROSPA_NΔ44-OspA_NΔ6 complex (see Supplementary Table S3).

**Figure 7:**
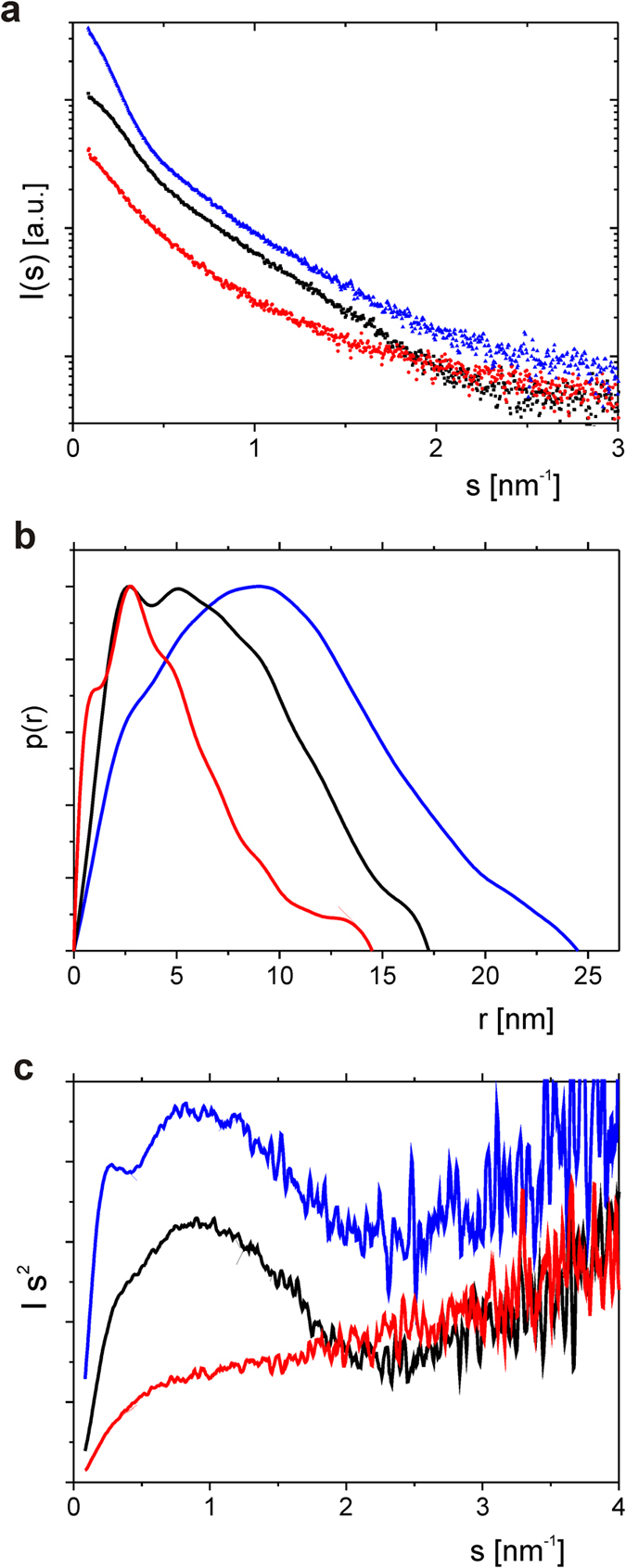
SAXS data on the TROSPA_NΔ44-OspA_NΔ6 complex. (**a**) Experimental X-ray scattering data plotted as a function of the scattering angle. (**b**) Pair-distance distribution functions. (**c**) Kratky plots. The red curve represents the data collected for TROSPA_NΔ44, the black curve represents the data collected for OspA_NΔ6 and the blue one represents the data collected for the TROSPA_NΔ44-OspA_NΔ6 complex.

To obtain more insight into the architecture of the TROSPA_NΔ44-OspA_NΔ6 complex, we also applied the SAXS method. The SAXS curves, p(r) functions and Kratky plots obtained for TROSPA_NΔ44, OspA_NΔ6 dimer and their complex are shown in Fig. 7. The p(r) function calculated for unbound TROSPA_NΔ44 contained several peaks, was asymmetric and exhibited a significantly gentler slope at higher r values, which indicated the highly elongated shape of this protein. The p(r) function obtained for OspA_NΔ6 contained several peaks and was less asymmetric than that of TROSPA_NΔ44, which indicated its slightly elongated shape. The p(r) function obtained for the complex was almost symmetric and contained a single peak. This shape suggests that the complex somewhat resembles a globular protein (a symmetric shape of p(r) and a single maximum are hallmarks of globular proteins)^29^,^34^. The Kratky plots obtained for both OspA_NΔ6 and the complex differ from the one generated for TROSPA_NΔ44 and resemble those obtained for the folded proteins. The deflection of the left arms of the parabolic curves suggests the multidomain character of OspA_NΔ6 and the complex^29^. The linearity of the Guinier plot is a prerequisite for further inferring from SAXS data^37^,^38^,^39^,^40^,^41^. Since Guinier plot for the complex was linear (see Supplementary Fig. S5), we considered the quality of our SAXS data is good enough to compute the biophysical parameters (M_m_, R_g_ and D_max_) of the complex and both TROSPA_NΔ44 and OspA_NΔ6 proteins. These parameters were determined from the Guinier extrapolation and the p(r) function^45^,^50^. The M_m_ value calculated for TROSPA_NΔ44 was approximately 13 kDa and corresponded to the M_m_ value calculated from its sequence (of 12.5 kDa). An experiment involving only OspA_NΔ6 indicated that it existed as a dimer in solution because the M_m_ calculated from the SAXS data was approximately 60 kDa, whereas the M_m_ calculated from its sequences was 32.37 kDa. This structure is supported by the Kratky plot, which suggests the multidomain character of OspA_NΔ6 (Fig. 7). The OspA dimer was not observed during previous structural studies of OspA that was devoid of the first 17 amino acid residues at its N-terminus^46^,^47^,^48^,^49^. Thus, OspA_NΔ6 forms a dimer in response to the presence of additional amino acids at its N-terminus (11 additional amino acids). The M_m_ calculated for the complex was 89 kDa, which indicates that one OspA_NΔ6 molecule binds one TROSPA_NΔ44 molecule (the M_m_ of the analogous complex calculated from the sequences is 82.28 kDa). In addition, the hydrated particle volume of the studied proteins was assessed using Porod volume calculations^50^. The volume calculated for unbound TROSPA_NΔ44 based on SAXS data was 43.07 nm^3^, and the volume calculated for the complex was 242 nm^3^. The volume of the OspA_NΔ6 dimer was 131.89 nm^3^. By comparison, the volume of the monomeric OspA devoid of the first 17 amino acids calculated based on its crystal structure was approximately 60 nm^3^. Altogether, these results indicate that recombinant TROSPA_NΔ44 can bind OspA_NΔ6 at a molar ratio of 2:2 and undergoes so-called folding upon binding.

## Discussion

TROSPA, a tick protein of unknown physiological function, is anchored in the membrane of the gut epithelium and serves as a receptor that binds bacterial surface proteins. The vast majority of experimental and computational data presented here indicate that the 121-amino acid outer membrane portion of the polypeptide chain of TROSPA from *I. ricinus* is intrinsically disordered. According to *in silico* disorder predictions, this protein lacks any regular structure, excluding the C-terminal portion that spans the last 25 amino acid residues (Fig. 1). These *in silico* predictions are consistent with the results of several independent experiments, which showed that TROSPA_NΔ44 is characterized by increased physicochemical parameter values (hydrodynamic volume, R_g,_ and D_max_), small secondary structure contents and a multi-conformer state. These features are all hallmarks of IDPs. The only aspect of TROSPA that was not typical of IDPs was its location on the charge-hydropathy plot; it clustered with ordered or globule-forming proteins^11^,^15^,^16^. However, we found that TROSPA shares no sequence similarity with any other known protein, whereas the charge-hydropathy plots considered here were created based on existing IDP sequence databases. Thus, the discrepancy between these plots and TROSPA features would be the consequence of the uniqueness of its amino acid sequence. In addition, Oldfield and coworkers showed that for some proteins, a charge-hydropathy plot tends to underestimate the protein’s propensity for disorder^15^, and which is supported by the experimental data presented here. Moreover, Das & Pappu suggested that inferring the sequence-ensemble of weak polyampholytes like TROSPA should include not only the charge-hydropathy relationship but also other considerations, such as the compositions of polar amino acids, the proline contents, and the presence of sequence stretches with preferences for specific secondary structures^16^.

Both the transiently and permanently structured motifs in IDPs are thought to be involved in ligand recognition and binding^18^,^19^,^29^,^37^,^38^,^39^,^40^,^41^,^51^,^52^. We monitored the structural changes of TROSPA_NΔ44 in response to changes in the pH, temperature, and concentration as well as the presence of its ligand. The CD measurements revealed that this protein can form transient secondary structural elements at pH 7.4 and a temperature of approximately 37 °C (Fig. 3, Supplementary Table S1). Interestingly, the EOM analysis revealed that the TROSPA_NΔ44 conformer population is multimodal in character, as evidenced by the presence of more compact subpopulations at higher concentrations (Fig. 5a,b, Supplementary Figs S3 and S4, Supplementary Table S2). These results are consistent with the results of the CD studies and indicate that TROSPA_NΔ44 contains transient secondary structural elements that become structured under conditions corresponding to the natural conditions of TROSPA-OspA recognition^1^,^30^.

An *in silico* analysis of the TROSPA_NΔ44 amino acid sequence based on different disorder predictors suggest the presence of a structured region at its C-terminus that spans amino acid residues 86–116 (Fig. 1). These findings corroborate CD measurements, which show that the permanent secondary structure content in TROSPA_NΔ44 reaches up to 20% (Fig. 3, Supplementary Table S1). Duplicated sequence motifs, including positions 2–25 and 54–77 of the TROSPA_NΔ44 polypeptide chain, were also observed (Fig. 1). Duplicated motifs in IDPs have been suggested to function as elastic linkers (entropic spacers) that connect more structured regions and might reflect the duplication-mediated evolution of these proteins^17^,^18^,^19^,^23^. Both our *in silico* predictions and experimental data suggest the presence of transient secondary structured motifs in TROSPA_NΔ44. The results of CD and SAXS obtained for the TROSPA_NΔ44-OspA_NΔ6 solution revealed that the complex is formed and the binding is accompanied by a further increase in the α-helical structure content of TROSPA_NΔ44 (Figs 6 and 7). The above results all suggest that the disordered polypeptide chain of TROSPA undergoes folding upon the binding of the ligand.

In our previous work, we tested a series of mutants containing substitutions that neutralize the total negative charge of TROSPA. We found that individual mutations slightly decreased but did not preclude the binding between TROSPA and its ligand OspA from *Borrelia garinii*^5^. These mutations were located outside the duplicated regions and predicted MoRFs; thus, they might be of minor importance for TROSPA-OspA binding. However, they may nonetheless influence the process of TROSPA folding by locally changing the attraction/repulsion between charged amino acid residues or the hydrophobicity^36^,^44^. Further detailed structural and functional studies are required to fully understand the dynamics and possible folding in response to the ligand binding of TROSPA. Native TROSPA is most probably subject to posttranslational modifications^2^, which might stiffen and elongate the unstructured polypeptide chain and enable pleiotropic interactions with IDP conformers^53^,^54^. By contrast, our earlier studies and the results obtained by other researchers indicate that posttranslational modifications of TROSPA are not necessary for its binding to OspA^2^,^5^.

TROSPA from *Ixodes* species binds to a group of OspA proteins from different *Borrelia* species^5^. Moreover, TROSPA from another hard tick, *Rhipicephalus microplus*, participates in the colonization of this tick by the protozoan pathogen *Babesia bigemina*^3^,^4^. *Ixodes* and *Ricephalus* TROSPA proteins share very high levels of homology (100% identity between the outer membrane parts of these proteins)^3^. Therefore, TROSPA might be a receptor that is used by at least two pathogens to colonize their vector. We showed that TROSPA is also an IDP that adapts its structure to the environment, and such proteins are usually able to bind many partners with high selectivity. The *I. scapularis* digestive system was recently shown to serve as a habitat for many nonpathogenic microorganisms that play a role in its proper functioning, and that these microorganisms positively influence the colonization of the tick by *Borrelia*^55^. Nevertheless, TROSPA may participate in interactions with beneficial microorganisms that dwell in the tick gut as well as with other pathogenic agents. The use of TROSPA for the immunization of animals has recently been proposed as a strategy to reduce the number of *Borrelia-* and *Babesia*-infected ticks^2^,^5^. Considering the observations described herein, this concept is of additional importance because a factor that efficiently binds to TROSPA could be used to restrict the spread of tick-borne diseases.

Finally, the TROSPA-OspA system described here represents a new and interesting model that can be used to study a wide spectrum of problems related to IDPs, especially in the context of host-pathogen interactions and inferring sequence ensembles. An important advantage of this system is the fact that OspA’s structure has been established^46^,^47^,^48^,^56^. Thus, our new model can be applied to protein folding studies because the rules governing this process are unclear and remain the Holy Grail of protein structural biology^25^.

## Methods

### Bioinformatics analyses

All bioinformatics analyses were performed using freely available online computational tools. A search for proteins with similar sequences was performed using the NCBI BLAST tool, and protein sequences were compared with the sequences included in the DisProt and Pfam databases^12^,^14^,^57^. The level of disorder of the protein structure was assessed using the MetaDisorder program, which is accessible via the GeneSilico MetaDisorder website. This program combines the results from 13 disorder prediction tools: DisEMBL (version 3), DISOPRED2, DISpro, Globplot, iPDA, IUPred (versions 2), Pdisorder, Poodle-s, Poodle-l, PrDOS, Spritz (versions 2), and RONN^13^. Additionally, three disorder prediction tools offered by the DisProt website were applied, PONDR-FIT, VSTX and VSL3^12^ (they are not included in the above-mentioned MetaDisorder program). The hydropathy values of TROSPA and TROSPA_NΔ44 were calculated according to the Kyte and Doolittle approximation^58^ using the ProtScale program available from the Expasy Bioinformatics Resource Portal, a window size of 5 amino acids and normalization of the values to a scale of 0 to 1. Next, the mean hydropathy was determined by calculating the sum of the normalized hydropathy values of all residues divided by the total number of residues in the protein. The NCPR was determined by calculating the net charge at pH 7.0 divided by the total number of residues. The FCR was calculated by dividing the percentage of charged residues by 100%^16^. The estimated M_m_ and pI were calculated according to the Compute pI/Mw program, which is available from the Expasy Bioinformatics Resource Portal. To search for the location of probable regions of ligand binding, the following MoRFs prediction online tools were applied: ANCHOR and MoRFpred^17^,^21^,^22^.

### Protein production and purification

TROSPA_NΔ44 and OspA_NΔ6 were expressed in *E. coli* and purified via affinity chromatography as previously described^5^. An additional purification step, SEC on a HiLoad 16/60 Superdex 200 prep grade column (GE Healthcare) equilibrated with buffer composed of 25 mM Tris-HCl, pH 8, 200 mM NaCl and 1 mM Tris (2-carboxyethyl) phosphine, yielded a homogenous fraction of protein. Finally, the protein was concentrated by ultra-filtration (Amicon Ultra-10,000 MWCO, Millipore), followed by ultra-filtration through 100,000 MWCO. The concentration of the protein in the filtrate was determined using a Nanodrop (Thermo Fisher Scientific) by measuring absorbance at 280 nm and considering the molar extinction coefficient and molecular mass of the protein. The monomeric state of the protein at each concentration was monitored by DLS measurements. To obtain the TROSPA_NΔ44 - OspA_NΔ6 complex, solutions of both proteins were mixed at molar ratio 1:1 and incubated for 5 min at room temperature.

### Assessment of molecular dimensions

The molecular mass-to-volume relationship of TROSPA_NΔ44 was initially assessed by SEC (as described above), using BSA, lysozyme and TEV protease as standards. SEC was performed under both native [in a buffer composed 25 mM Tris-HCl, pH 8, 200 mM NaCl and 1 mM Tris (2-carboxyethyl) phosphine] and denaturing conditions [in a buffer composed 25 mM Tris-HCl, pH 8, 200 mM NaCl, 1 mM Tris (2-carboxyethyl) phosphine and 8 M urea] at 20 °C. The fractionated proteins were identified by SDS-PAGE. The M_m_ of TROSPA_NΔ44 was also determined using Ultraflextreme MALDI Tof/Tof mass spectrometry (Bruker Daltonics). The polydispersity and R_h_ of the protein were determined by dynamic light scattering (DLS) using a Zetasizer μV (Malvern). The measurements were performed immediately after the concentration and filtration steps using a 4-μl sample in a 1-cm path-length quartz cuvette (Hellma QS 105.231). DLS measurements were also employed to estimate the thermostability of the protein samples and the changes in the R_h_ at different temperatures. The heating rate was 1 °C min^−1^, data were collected every 2 °C, and the measurements began after a 5-min equilibration at each step. Thirteen measurements were taken for each temperature (ranging from 4 °C to 80 °C).

### CD measurements

CD spectra were collected on a J-815 CD spectrometer (JASCO) equipped with a Peltier-thermostated cell holder. Protein solution (80 μg/ml) in buffer consisting of 25 mM sodium phosphate and 100 mM NaF at pH 6, 6.4, 7, 7.4 or 8 was analyzed in a 0.2-cm quartz cuvette (Hellma 100-QS). Each CD spectrum was generated based on 3 scans in continuous scanning mode, with a scanning speed of 50 nm min^−1^, a 1-nm bandwidth, a 0.5-nm data pitch and a data integration time of 1 sec. Data were collected at wavelengths ranging from 185 to 350 nm when collecting a regular spectrum or at wavelengths ranging from 185 to 260 nm for the thermal melt analysis. The spectra were analyzed using CONTIN/LL (available on the DichroWeb server) after processing in the Jasco Spectra Manager software using the Savitzky-Golay tool with a smoothing window of 20 points (Fig. 3). The normalized root mean square deviation (NRMSD) for each CD spectrum analysis was less than 0.1. CD data are presented in terms of ellipticity in millidegrees (mdeg) or as the mean residue ellipticity [θ]_MRW_ in deg cm^2^ dmol^−1^. The thermal melting of the protein was monitored at temperatures ranging from 5 to 80 °C. The heating rate was 1 °C min^−1^, and data were collected every 2 °C.

### SAXS measurements

SAXS patterns were collected at beamlines: P12 of the Petra III storage ring at the DESY (Deutsches Elektronen-Synchrotron) in Hamburg, Germany and I911-4 beamline at the MAX Lab in Lund, Sweden. Twenty-microliter samples of protein (TROSPA_NΔ44 concentration range 1–12 mg/ml, OspA_NΔ6 concentration 4 mg/ml) and of the corresponding matching buffer (25 mM Tris-HCl, pH 8, 200 mM NaCl and 1 mM Tris (2-carboxyethyl) phosphine) were analyzed. All data were collected at 15 or 20 °C. SAXS data were collected over the s range of 0.0088–5 nm^−1^ (DESY) or 0.01–4.5 (MAX IV), and overlays of the merged data sets were used to detect concentration-dependent scattering in the lowest s region. All SAXS data were processed using ATSAS suite^59^,^60^. The integration, scaling, and buffer subtraction were accomplished using the PRIMUS program^29^,^33^,^59^,^60^,^61^,^62^. The resultant curves were used for all further calculations and reconstructions. The M_m_ values of analyzed proteins were calculated by comparing the extrapolated *I*(0) values with that of a standard bovine serum albumin sample using equation (1):


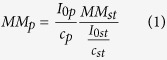


where *MM*_*p*_ and *MM*_*st*_ are the molecular weights of studied and standard protein, *I*_*0p*_ and *I*_*0st*_ are the scattering intensities at a zero angle of studied and standard protein, and *c*_*p*_ and *c*_*st*_ are the concentrations of studied and standard protein, respectively. *R*_*g*_ within the range of the Guinier approximation *s*_*max*_ *R*_*g*_ < 1.3 was evaluated according to equation (2):


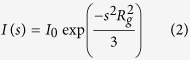


The R_g_, D_max_ and p(r) functions were evaluated using GNOM^33^. EOM was used to obtain information about the possible structural heterogeneity and biophysical parameters of TROSPA_NΔ44^29^,^44^. We used a pool of 10000 independent models of TROSPA_NΔ44 based on sequence and structural information. No rigid body was used as the input, and the complete random configurations of the α-carbon trace were created based on the sequence. Once the pool generation was complete, the appropriate subsets of configurations that fit the experimental SAXS data were selected by a genetic algorithm. Finally, selected models should best fit the experimental curves, I(s). The discrepancies between the experimental and calculated curves (defined by the χ^2^ coefficient in the EOM software) were close to 1, which is an acceptable value.

## Additional Information

**How to cite this article**: Urbanowicz, A. *et al*. Tick receptor for outer surface protein A from *Ixodes ricinus* – the first intrinsically disordered protein involved in vector-microbe recognition. *Sci. Rep.* **6**, 25205; doi: 10.1038/srep25205 (2016).

## Supplementary Material

Supplementary Information
