# Supplementary material for: Tick receptor for outer surface protein A from Ixodes ricinus — the first intrinsically disordered protein involved in vector-microbe recognition
Source: Sci Rep. 2016 Apr 26;6:25205. doi: 10.1038/srep25205 (PMC4844993; doi:10.1038/srep25205)
Supplement: Supplementary Information [file srep25205-s1.pdf]

Tick receptor for outer surface protein A from *Ixodes ricinus* — the first intrinsically disordered protein involved in vector-microbe recognition

Anna Urbanowicz[1, +], Dominik Lewandowski[1, +], Kamil Szpotkowski[1], and Marek Figlerowicz[1, 2, *]

[1]Institute of Bioorganic Chemistry, Polish Academy of Sciences, Poznan, 61-704, Poland

[2]Institute of Computing Science, University of Technology, Poznan, 60-965, Poland

[*] marekf@ibch.poznan.pl

[+]These two authors contributed equally to this work

Supplementary information

pH 6					pH 6.4					pH 7					pH 7.4					pH 8				
**°C**	**α**	**β**	**T**	**D**	**°C**	**α**	**β**	**T**	**D**	**°C**	**α**	**β**	**T**	**D**	**°C**	**α**	**β**	**T**	**D**	**°C**	**α**	**β**	**T**	**D**
**5**	4%	8%	8%	80%	**5**	5%	2%	5%	88%	**5**	8%	7%	12%	73%	**5**	10%	19%	15%	56%	**5**	4%	7%	7%	82%
**10**	4%	9%	7%	81%	**10**	6%	5%	4%	85%	**10**	7%	5%	10%	78%	**10**	10%	22%	17%	51%	**10**	5%	4%	5%	86%
**15**	5%	8%	7%	80%	**15**	5%	3%	5%	88%	**15**	8%	12%	14%	66%	**15**	8%	14%	16%	62%	**15**	4%	6%	6%	84%
**20**	6%	9%	8%	78%	**20**	6%	5%	6%	84%	**20**	9%	9%	13%	70%	**20**	10%	19%	16%	55%	**20**	7%	7%	7%	78%
**25**	6%	10%	9%	76%	**25**	6%	3%	6%	85%	**25**	17%	5%	13%	65%	**25**	10%	18%	16%	56%	**25**	8%	9%	7%	77%
**30**	6%	10%	8%	76%	**30**	6%	6%	6%	82%	**30**	9%	7%	13%	72%	**30**	8%	18%	18%	56%	**30**	5%	3%	8%	84%
**35**	7%	11%	10%	73%	**35**	6%	4%	7%	83%	**35**	9%	9%	13%	69%	**35**	16%	17%	16%	51%	**35**	7%	12%	6%	76%
**40**	7%	11%	9%	73%	**40**	7%	6%	7%	81%	**40**	8%	7%	13%	72%	**40**	15%	16%	16%	53%	**40**	8%	10%	7%	75%
**45**	7%	11%	10%	72%	**45**	6%	5%	7%	82%	**45**	17%	5%	14%	65%	**45**	13%	12%	17%	58%	**45**	6%	4%	8%	83%
**50**	7%	11%	9%	73%	**50**	6%	3%	4%	87%	**50**	16%	5%	12%	67%	**50**	13%	14%	17%	56%	**50**	7%	9%	6%	79%
**55**	7%	8%	8%	77%	**55**	6%	6%	4%	84%	**55**	16%	6%	12%	66%	**55**	6%	5%	9%	80%	**55**	6%	5%	9%	80%
**60**	8%	11%	9%	72%	**60**	6%	5%	6%	83%	**60**	17%	3%	12%	68%	**60**	4%	4%	6%	86%	**60**	4%	4%	6%	86%
**65**	7%	10%	10%	74%	**65**	7%	4%	5%	84%	**65**	7%	2%	6%	85%	**65**	7%	4%	7%	82%	**65**	7%	4%	7%	82%
**70**	8%	11%	8%	74%	**70**	6%	3%	6%	85%	**70**	15%	1%	6%	78%	**70**	7%	2%	5%	87%	**70**	7%	2%	5%	87%
**75**	7%	7%	9%	78%	**75**	7%	7%	6%	80%	**75**	7%	0%	7%	86%	**75**	5%	2%	4%	88%	**75**	5%	2%	4%	88%
**80**	7%	7%	7%	79%	**80**	6%	4%	4%	86%	**80**	7%	0%	8%	84%	**80**	5%	5%	2%	88%	**80**	5%	5%	2%	88%

Supplementary Table S1. The secondary structure of TROSPA_NΔ44 as a function of pH and temperature. The secondary structure content was estimated from CD spectra using the CONTIN/LL algorithm (reference set 7). α – α helices, β – β strands, T- turn, D- disordered region. The results obtained over temperature range of 35-40°C (corresponding to natural conditions of TROSPA-OspA binding) are marked with green. Normalized root mean square deviation (NRMSD) for each CD spectrum analysis was less than 0.1.

Concentration	Distribution (EOM)	R_g (EOM)	R_g (EOM) averaged	D_{max} (EOM)	R_g (Guinier)	M_m
1 mg/ml	36%	3.59nm	4.15nm	10.26nm	3.73±0.012	12,789Da
	64%	4.47nm		12.10nm		
3mg/ml	67%	4.04nm	4.05nm	11.49nm	3.7±0.009	12,832Da
	22%	4.26nm		11.36nm		
	11%	3.66nm		9.84nm		
6mg/ml	69%	3.74nm	3.80 nm	12.78nm	3.65±0.008	12,889Da
	31%	3.92nm		9.89nm		
9mg/ml	8%	3.74nm	3.89 nm	11.23nm	3.62±0.013	12,910Da
	92%	3.92nm		11.40nm		
12mg/ml	25%	3.54nm	3.80nm	9.25nm	3.58±0.011	12,965Da
	25%	3.50nm		10.43nm		
	37%	3.95nm		11.37nm		
	13%	4.46nm		11.25nm		

Supplementary Table S2. SAXS-derived parameters for TROSPA_NΔ44. Processing of SAXS data by EOM revealed multimodal distribution of conformers of TROSPA_NΔ44 regarding the values of both R_g and D_{max}. Increasing protein concentration is coupled with decreases in the R_g (parameters determined both by EOM and by Guinier approximation). M_m was calculated from I_0 as described in Methods section. M_m calculated from the amino acid sequence was 12.5 kDa.

	α	β	T	D
**TROSPA_NΔ44, pH 8**	7%	12%	6%	76%
**TROSPA_NΔ44, pH 7.4**	16%	17%	16%	51%
**OspA_NΔ6, pH 7.4**	10%	46%	19%	25%
**TROSPA_NΔ44-OspA_NΔ6 complex, pH 7.4**	34%	46%	12%	8%

Supplementary Table S3. The comparison of secondary structure content of TROSPA_NΔ44, OspA_NΔ6 and TROSPA_NΔ44-OspA_NΔ6 complex at 37°C. The secondary structure content was estimated from the CD spectra using CONTIN/LL algorithm. α – α helices, β – β strands, T- turn, D- disordered region. Normalized root mean square deviation (NRMSD) for each CD spectrum analysis was less than 0.1.

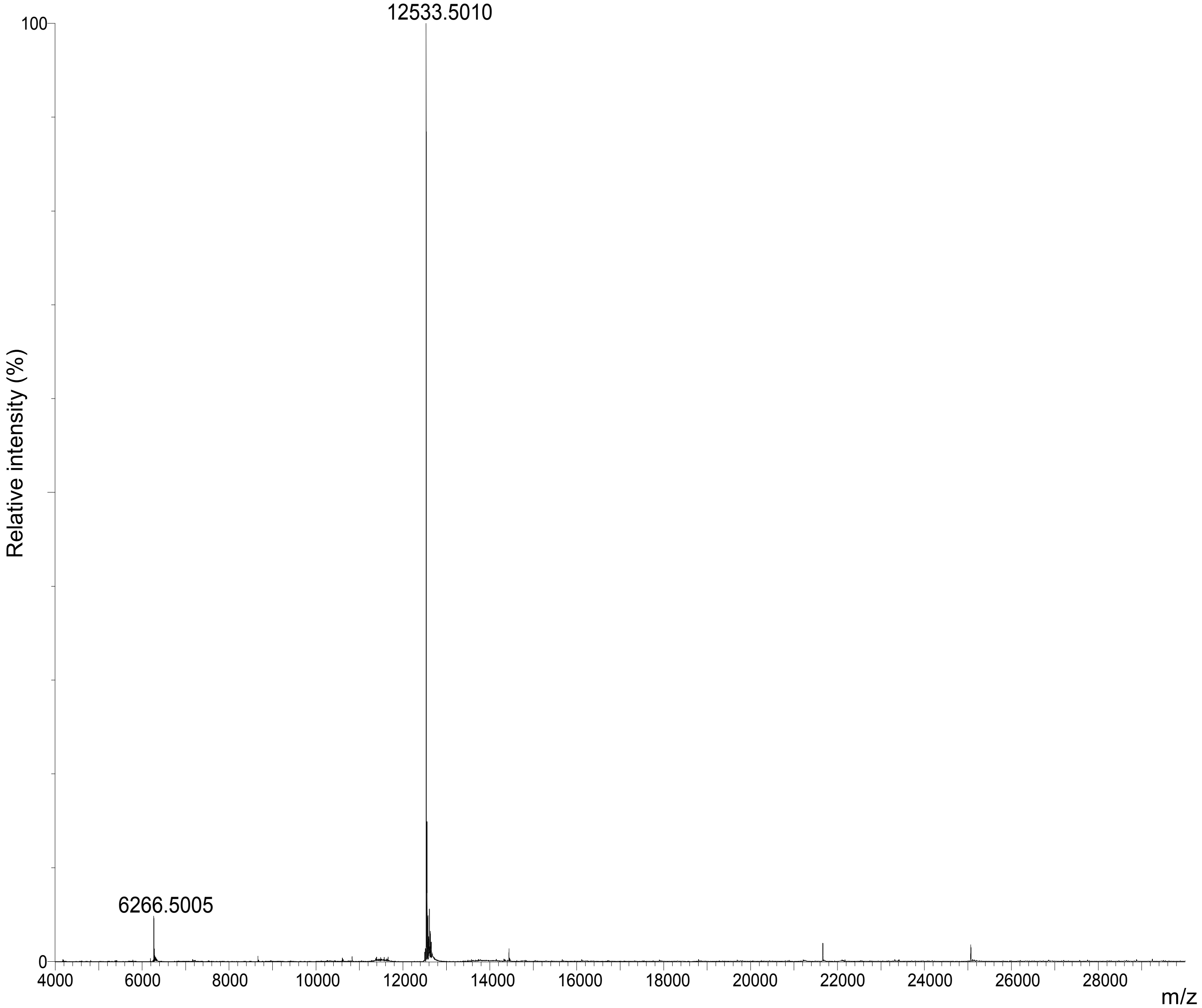

Supplementary Figure S1. MALDI-TOF mass spectrum of TROSPA_NΔ44. Doubly (at m/z 6266.5005) and singly (at m/z 12533.5010) charged ions are visible.

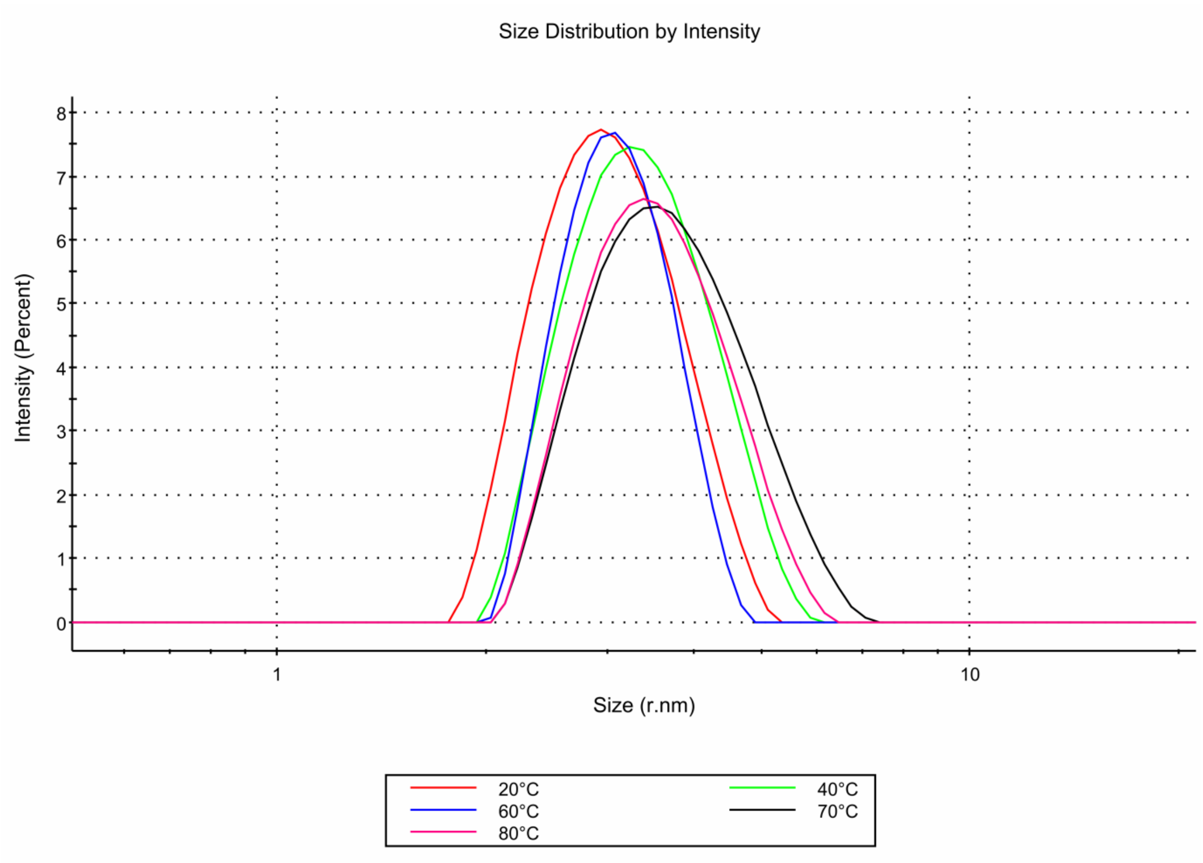

Supplementary Figure S2. Determination of R_h of TROSPA_NΔ44 over a temperature range of 20–80 °C by DLS.

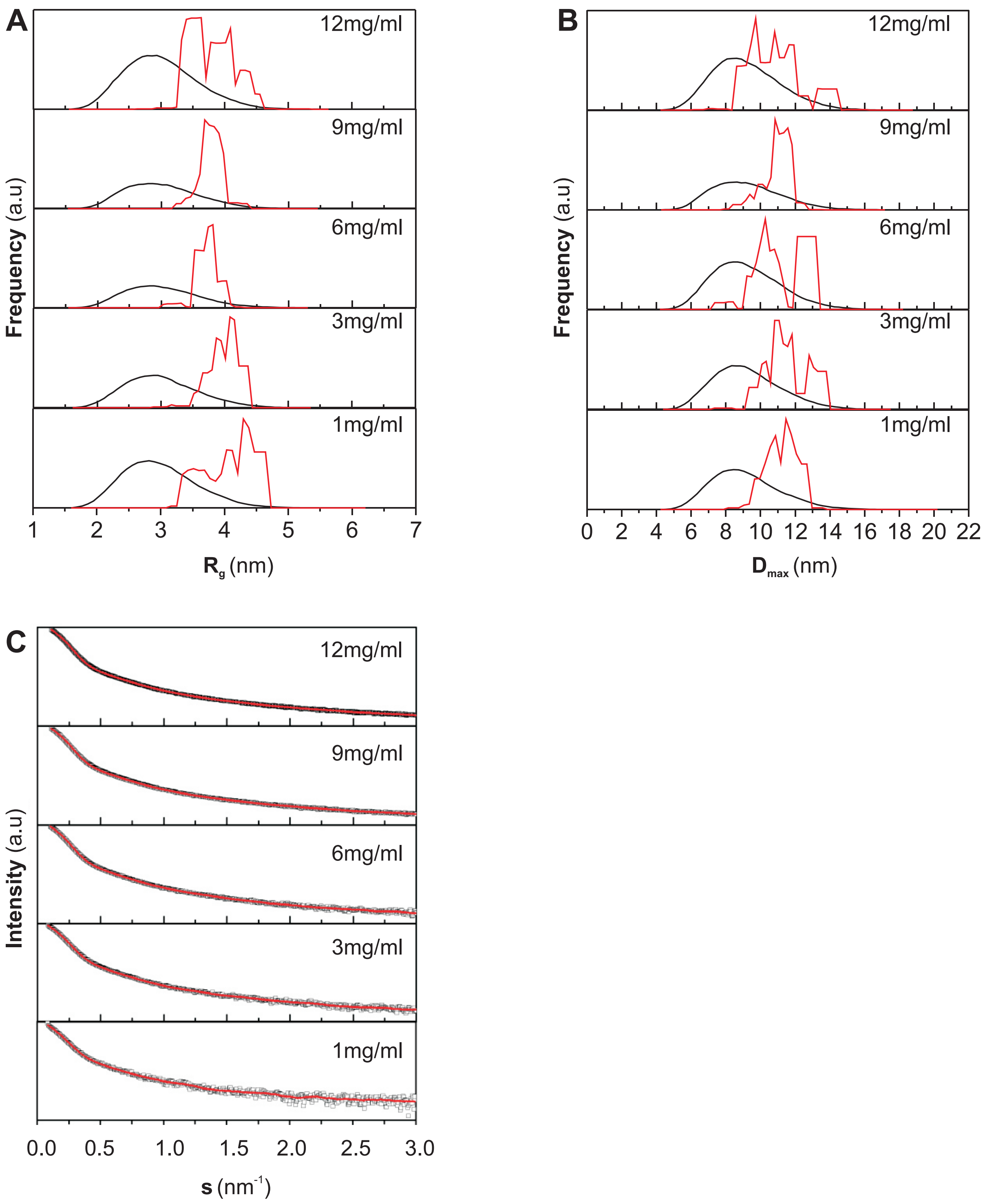

Supplementary Figure S3. SAXS data processed by EOM for TROSPA_NΔ44 at pH 8 at five different concentrations: 1, 3, 6, 9 and 12 mg/ml regarding both R_g (A) and D_{max} (B). R_g and D_{max} distributions for TROSPA_NΔ44 plotted as functions of frequency (arbitrary units) indicate the multimodal character of the protein population. The red curves represent the distribution that best fits the SAXS data. The black curves represent the theoretical distribution of R_g and D_{max} values in a situation when the entire polypeptide chain would be able to form all the possible conformations. **EOM fits to the experimental scattering curves, I(s) (C).** The discrepancies between the experimental (black squares) and calculated (red line) curves (defined by the χ^2 coefficient in the EOM software) were close to 1.

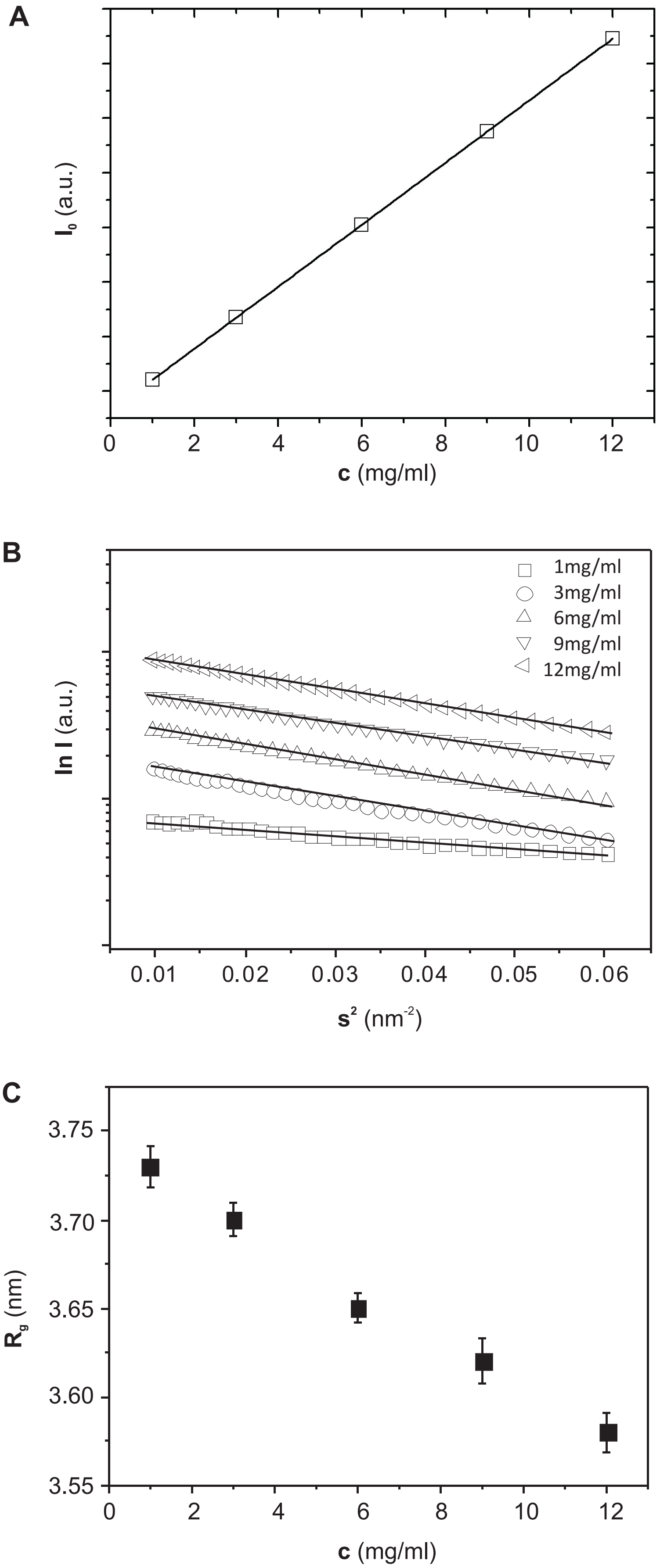

Supplementary Figure S4. Experimental SAXS data for TROSPA_NΔ44 at pH 8 at five different concentrations: 1, 3, 6, 9 and 12 mg/ml. Linear I_0 relationship with protein concentration (A), linear Guinier plot (B) and R_g values determined by Guinier approximation (C). The error bars represent standard deviation from linear fits to the Guinier curves.

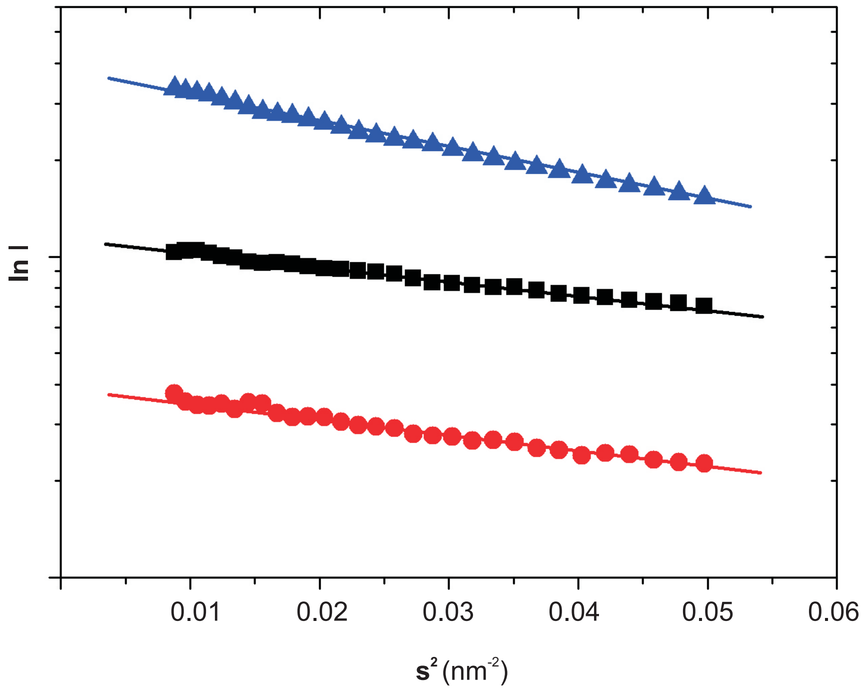

Supplementary Figure S5. Guinier plots for TROSPA_NΔ44 (red circles), OspA_NΔ6 (black squares) and for TROSPA_NΔ44-OspA_NΔ6 complex (blue triangles).
